# Survey of Antibiotic-producing Bacteria Associated with the Epidermal Mucus Layers of Rays and Skates

**DOI:** 10.3389/fmicb.2017.01050

**Published:** 2017-07-05

**Authors:** Kim B. Ritchie, Melbert Schwarz, Joseph Mueller, Valeri A. Lapacek, Daniel Merselis, Catherine J. Walsh, Carl A. Luer

**Affiliations:** ^1^Department of Natural Sciences, University of South Carolina Beaufort, BeaufortSC, United States; ^2^Mote Marine Laboratory, SarasotaFL, United States; ^3^Department of Biological Sciences, University of Quebec at Montreal, MontrealQC, Canada; ^4^Department of Biological Sciences, Florida International University, MiamiFL, United States

**Keywords:** antibiotic producing bacteria, antibacterial screening, pathogens, epidermal mucus, stingray, skate, beneficial bacteria

## Abstract

Elasmobranchs represent a distinct group of cartilaginous fishes that harbor a remarkable ability to heal wounds rapidly and without infection. To date very little work has addressed this phenomenon although it is suggested that antibiotic capabilities associated with epidermal surfaces may be a factor. The study of benefits derived from mutualistic interactions between unicellular and multicellular organisms is a rapidly growing area of research. Here we survey and identify bacterial associates of three ray and one skate species in order to assess the potential for antibiotic production from elasmobranch associated bacteria as a novel source for new antibiotics.

## Introduction

Marine bacteria are genetically and metabolically diverse, capable of producing a wide range of chemical compounds, and are known to establish symbioses with a range of marine organisms (Bhatnagar and Kim, 2010; Sobhana, 2015). As a result, there is a growing interest in mutually beneficial associations between microbes and their hosts (Zamioudis and Pieterse, 2012). Increasing studies illustrate that bacteria produce chemical compounds that were previously ascribed to marine hosts, providing bioactive compounds for utilization in host defenses (Chau et al., 2013; Abdelmohsen et al., 2014). The study of host-associated microbes also provides a unique avenue for the search for novel bioactive compounds that could be utilized in biomedical applications (Vasanthabharathi and Jayalakshmi, 2013).

Studies in corals have shown that antibiotic activity is present in the surface mucus of healthy corals (Ritchie, 2006) Furthermore, greater than 20% of bacteria isolated from the mucus of the elkhorn coral, *Acropora palmata*, demonstrated antibiotic activity against a range of pathogenic test strains and 8% were specifically active against a pathogen that causes disease in this species (Ritchie, 2006). These results suggest that organisms may derive some of their immunity from probiotic bacteria associated with their surface mucus layers.

Aqueous protein extracts of the epidermal mucus layers of several finfishes have been investigated as a source of innate immunity and have demonstrated broad-spectrum antibiotic activity (John and Patterson, 2011). Similar extracts from the mucus layers of Atlantic cod, (Bergsson et al., 2005) and hagfish (Subramanian et al., 2008) have also demonstrated antibiotic activity against several common infectious pathogens, further illustrating that innate immunity is an important immune function for aquatic organisms. As part of their normal life activities, notably associated with regular mating events, aggressive behavior, predation, and anthropogenic encounters, sharks and their skate and ray relatives routinely sustain and recover from wounds penetrating the epidermal and dermal layers of the skin (Stevens, 1974; Pratt, 1979; Hoyos-Padilla et al., 2013). Such traumatic wounds heal completely and apparently without infection, even when continuously exposed to an environment rich in pathogens (Towner et al., 2012; Chin et al., 2015). With the regular occurrence of fresh and well-healed wounds on stingrays (Snelson et al., 1988; Kajiura et al., 2000) it is possible that their epidermal mucus may serve an innate immune function to account for the absence of wound-related infection.

Several studies have already investigated the possibility of such an immune function and found that chemical extracts of stingray epidermal mucus display antibiotic activity. Vennila et al. (2011) demonstrated that acidic extracts from the epidermal mucus of *Dasyatis sephen*, the cowtail stingray, and *Himantura gerrardi*, the sharpnose stingray, displayed peptide derived antimicrobial action, while Conceição et al. (2012) identified protein based antibiotic activity in the epidermal mucus of *Potamotrygon henlei*, the bigtooth river stingray. In addition, aqueous spine extracts of *Himantura imbricata*, the scaly stingray, demonstrated broad spectrum antibiotic activity (Kalidasan et al., 2014). In a microbial based, human health oriented study, it was found that the epidermal mucus of the ocellate river stingray, *Potamotrygon motoro*, harbored a number of bacteria that were toxic to human epithelial cells (Domingos et al., 2011). However, no studies have isolated and characterized antibiotic-producing bacteria associated with the epidermal mucus layers of stingrays.

The purpose of this study was to isolate and identify antibiotic producing bacterial strains from the epidermal mucus of skates and rays that may serve a potential probiotic role against wound infections as well as provide a novel source for antibiotics. With the realization that human wound infection pathogens are adapting to existing antibiotic drugs and becoming increasingly resistant to treatment over time, the United States Department of Defense is interested in developing new antibiotic compounds to treat their wounded warriors. As a result of the recurring observation of infection-free healing in elasmobranch fishes, the data presented here are part of a study funded by the Department of Defense to establish the feasibility of identifying novel compounds from stingray epidermal mucus with potential application in treating wound infection pathogens and promoting wound healing.

## Materials and Methods

### Experimental Animals

Animals used as sources of epidermal mucus included cownose ray, *Rhinoptera bonasus*, Atlantic devil ray, *Mobula hypostoma*, marine and freshwater Atlantic stingrays, *Dasyatis sabina*, and clearnose skate, *Raja eglanteria*. All marine species are inhabitants of Gulf of Mexico waters off the coast of Sarasota, FL, United States, at various times of the year. Freshwater *D. sabina* are permanent inhabitants in lakes near Orlando, FL, United States.

### Specimen Collection

Cownose rays and devil rays were collected passively by surrounding schools in shallow water with a seine net and transferring individual rays with dip nets to an onboard live-well. Marine Atlantic stingrays were captured in shallow nearshore water using cast nets, while deeper water clearnose skates and freshwater Atlantic stingrays were collected using baited set lines. Cownose rays, devil rays, and freshwater Atlantic stingrays were sampled at time of capture and released unharmed, while marine Atlantic stingrays and clearnose skates were returned to the laboratory where samples were collected. All animals were collected following guidelines specified in Special Activities Licenses issued by the Florida Fish and Wildlife Conservation Commission. Numbers of individuals from each species are as follows: Freshwater *D. sabina*, *n* = 3 (1 male; 2 females), Marine *D. sabina*: *n* = 12 (3 males; 9 females), *M. hypostoma*: *n* = 30 (20 males; 10 females), *R. bonasus*: *n* = 74 (27 males; 47 females), *R. eglanteria*; *n* = 3 (all female).

### Mucus Collection

**FIGURE 1:**
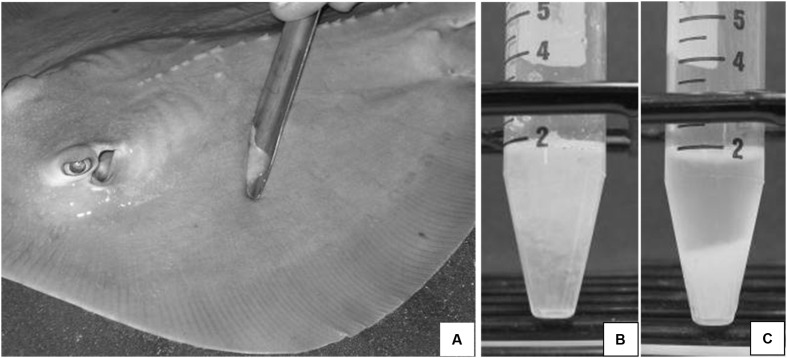
**(A)** Epidermal mucus is collected by passive scraping of the pectoral fin surface of an Atlantic stingray, *Dasyatis sabina* with a sterile scoopula. Fresh mucus **(B)** can be separated into an aqueous supernatant and a mucus pellet **(C)** following centrifugation at 2,600 × *g* for 20 min at 4°C.

Epidermal mucus was sampled from individual rays and skates by sterile seawater surface rinses followed by passive scraping of the pectoral fin surfaces with a sterile scoopula and transferred to sterile culture tubes. Mucus was separated by centrifugation (2,600 × *g*, 30 min, 4°C) into an aqueous supernatant and a viscous pellet (**Figure 1**).

### Bacterial Isolation

Aliquots (100 μL) of freshly collected mucus pellets and aqueous supernatants were serially diluted in sterile seawater (ranging in concentration relative to the original sample from 1:10 to 1:1,000,000). One hundred microliters of each dilution was plated onto marine agar (Sigma Chemical Co) with sterile 2 mm ColiRoller glass beads. Cultures were grown at 25°C for 3–5 days for development of bacterial colonies. Bacterial colonies exhibiting a unique morphology were sub-cultured 3 times to purify under the same set of growth conditions. Purified isolates were cryopreserved at -80°C in 25% glycerol in marine broth and stored in 96-well microtiter plate culturable libraries for antibiotic screening. Most of the mucus-associated bacteria were isolated from the mucus pellet fraction rather than the supernatant.

### Antibacterial Screening

Isolated colonies were analyzed for antibacterial properties against a number of pathogenic and non-pathogenic test strains using an agar overlay assay. Purified elasmobranch bacterial libraries were inoculated onto single well plates (VWR) of 1.5% marine agar using a 96-prong well inoculator (V&P Scientific, Inc.). Library plates were allowed to grow at 25°C for 2 days and were UV- irradiated for 30 min to prevent further growth and cross-contamination of isolates. Test strains were grown overnight at 25°C (marine pathogens) or 37°C (human pathogens) in 2 mL of strain specific culture broth and included *Bacillus subtilis* (ATCC 6633 Km resistant), *Enterococcus faecalis* (ATCC 29212), *Vancomycin-resistant Enterococcus* (VRE, ATCC MP-1), *Methicillin-sensitive Staphylococcus aureus* (MSSA, ATCC 29213), *Methicillin-resistant S. aureus* (MRSA, ATCC 43300), *Escherichia coli* O157 *Serratia marcescens* PDL100 and *Vibrio shilonii* BAA-91. Aliquots of each broth culture were inoculated into 0.8% agar containing marine broth, Luria broth or tryptic soy broth, depending on the test strain. Elasmobranch library plates were overlaid with approximately 10 mL of inoculated agar. Following overnight growth, plates were analyzed for antibacterial activity and zones of inhibition were measured in millimeters.

### Antibacterial Compound Characterization (Blood Agar and Proteinase K Assays)

A subset of antibiotic-producing isolates, chosen based on an ability to inhibits the growth of a varying range of pathogens as well as their stability upon repeated subculturing, was further characterized by testing for blood lysis potential and production of antimicrobial peptides. Eleven *R. bonasus* isolates displaying broad spectrum antibacterial activity in the initial screening process were inoculated onto 1.5% marine agar plates containing 5% sheep blood by volume. Following overnight growth, these strains were analyzed for the ability to lyse red blood cells by identifying and measuring zones of hemolysis (clearing zones) in millimeters. The antibacterial compounds produced by these isolates were further characterized by testing antibacterial activity in the presence of Proteinase K. Overlays were carried out as described with the addition of 100 μg/mL of Proteinase K to the test strain-appropriate 0.8% agar. Following overnight growth, zones of pathogen growth inhibition were identified and measured in millimeters. Results from this assay were then compared to initial overlay assays to determine which strains retained antibacterial properties in the presence of Proteinase K.

### Phylogenetic Characterization of Bacterial Strains

DNA was extracted from pure cultures of all antibiotic-producing isolates from stingrays using a Power Soil DNA Extraction Kit (Mo Bio, Inc.). The 16S rRNA genes were amplified using a 25 uL polymerase chain reaction (PCR) mixture as follows: 12.5 μL of Taq master mix (Qiagen), 1 μL of 0.5 μg/mL bovine serum albumin, 8.5 μL of molecular grade water, 1.0 μL (50 ng) of template DNA and 1 μL (10 μM) each of forward oligonucleotide primer U9F (5′-GAGTTTGATYMTGGCTC) and reverse primer U1502R (5′-GYTACCTTGTTACGACTT; Weidner et al., 1996). Cycling conditions included an initial denaturation at 94°C for 120 s followed by 35 rounds of 94°C for 80 s, 54°C for 60 s, and 72°C for 90 s with a final 72°C extension for 180 s. PCR products were identified via 1% agarose gel electrophoresis, visualized on an AlphaImager 3300, and purified using a Qiagen PCR purification kit (Qiagen, Inc). DNA from purified PCR samples was sequenced at the UIUC Core Sequencing Facility, University of Illinois, Urbana-Champaign. Consensus sequences of forward and reverse strands generated through sequencing were analyzed using the GenBank Basic Local Alignment Search Tool (BLAST) to determine percent similarity to other strains in the international bacteria sequence database (Altschul et al., 1997).

### Phylogenetic Tree of Antibiotic-Producing Bacteria

A phylogenetic tree was constructed using QIIME (Caporaso et al., 2010b). Consensus sequences for each strain found in **Table 2**, as well as four reference sequences from GenBank (*Pseudoalteromonas tunicata* strain D2: Z2552, *B. circulans*: FJ581445, *V. coralliilyticus*: HM771346, and *Photobacterium halotolerans*: AY551089) were aligned with each other with the function align_seqs.py in QIIME using the PyNAST method (Caporaso et al., 2010a). Following the initial alignment, the QIIME function filter_alignment.py was used to remove gaps in the alignment shared by all sequences. Using this alignment, the phylogeny was created using FastTree 2 (Price et al., 2010), the default method utilized by the QIIME function make_phylogeny.py. The FastTree method is an approximately maximum-likelihood method in which a skeleton tree is created, using a neighbor-joining algorithm, to gain an initial topography which is subsequently improved using maximum-likelihood rearrangements. The resulting phylogeny is unrooted by default. The phylogenetic tree was visualized in R using the package ggtree (Yu et al., 2016).

### GenBank Accession Numbers

16S rRNA gene sequences generated were entered into the GenBank world-wide database (NCBI) under accession numbers KP713443-KP713670.

### Ethics Statement

This study was carried out in strict accordance with the recommendations in the Guide for the Care and Use of Laboratory Animals of the National Institutes of Health and outlined in Mote Marine Laboratory’s Animal Welfare Assurance (A4219-01). All experimental protocols were approved by Mote Marine Laboratory’s Institutional Animal Care and Use Committee (IACUC) and by the US Army Medical Research and Materiel Command (USAMRMC) Animal Care and Use Review Office (ACURO). All biological samples were obtained passively with minimal discomfort and no animals died or were euthanized during the project.

## Results

During this study, 1860 bacteria were isolated from the epidermal mucus of three stingray species and one skate species (**Table 1**). All bacterial isolates were screened for their ability to produce antibacterial compounds that inhibit the growth of a range of pathogenic test strains. Three hundred and eleven of these isolates demonstrated activities against one or more test strains (**Table 1**). Of these 311, 57 produced either broad-spectrum antibiotics or activities against VRE or MRSA only (**Table 2**).

**Table 1:** Compiled stingray and skate antibacterial screening data.

Species	Common name	Bacterial isolates	# Isolates with antimicrobial activity	% Showing activity
*Dasyatis sabina* (F)	Atlantic stingray	96	9	9.4
*Dasyatis sabina* (M)	Atlantic stingray	323	60	18.6
*Rhinoptera bonasus*	Cownose ray	960	200	20.8
*Mobula hypostoma*	Devil ray	193	35	18.1
*Raja eglanteria*	Clearnose skate	288	7	2.4
	**Totals**	**1,860**	**311**	**16.7**


**Table 2:** List of representative bacteria isolated from the mucus of rays.

Strain	Bp	% ID	Strain identification	Isolate #	AB spectrum (ZI)	Accession #
**Freshwater Atlantic stingray, *Dasyatis sabina***						
845A3	780	99	*Microbacterium* sp.		*B. subtilis* (1.0)	KP713518
845D5	847	99	*Stenotrophomonas* sp.		*E. coli* (1.5)	KP713525
845E11a	926	99	*Pseudomonas stutzeri*		*E. coli* (1.5)	KP713564
845E11b	902	99	*Pseudomonas putida*	4	*E. coli* (1.5)	KP713565
845E4	539	100	*Psychrobacter pacificensis*		*E. coli* (1.0)	KP713562
845E9	896	100	*Bacillus cereus*		*E. coli* (1.0); MRSA (2.5); MSSA (1.5); VRE (2.5)	KP713528
845F2	879	99	*Pseudomonas* sp.		*E. coli* (0.5)	KP713566

**Marine Atlantic stingray, *Dasyatis sabina***						

846B4	839	99	*Photobacterium damselae*	2	*B. subtilis* (1.0)	KP713568
846C2	922	99	*Vibrio harveyi*	2	*B. subtilis* (1.0)	KP713569
846C5	939	99	*Photobacterium* sp.		*B. subtilis* (1.0)	KP713480
846F8	1003	99	*Vibrio* sp.	2	*B. subtilis* (1.0)	KP713572

**Atlantic devil ray, *Mobula hypostoma***						

809A8	844	99	*Vibrio* sp.		VRE (2.0)	KP713464
809B1	893	99	*Pseudoalteromonas* sp.	3	*B. subtilis* (1.0)	KP713465
809B6	962	99	*Pseudoalteromonas* sp.	4	*V. shilonii* (1.0)	KP713468
810E4	937	98	*Vibrio* sp.	10	*B. subtilis* (0.1)	KP713446
810F6	976	99	*Alteromonas* sp.		*B. subtilis* (0.5)	KP713449
810G1	967	98	*Vibrio* sp.		MRSA (0.1); *B. subtilis* (0.2)	KP713451

**Cownose ray, *Rhinoptera bonasus***						

803A6-1	880	99	*Exiguobacterium* sp.	3	*B. subtilis* (0.2)	KP713613
803B11	787	99	*Pseudoalteromonas* sp.		*B. subtilis* (1.75)	KP713582
803B6-3	870	99	*Bacillus* sp.	4	MRSA (0.3); MSSA (1.0); *B. subtilis* (3.5)	KP713633
803B8-1	889	99	*Exiguobacterium* sp.		MRSA (0.5); MSSA (0.2); *B. subtilis* (0.3)	KP713634
803B8-2	890	99	*Bacillus* sp.	2	MRSA (0.5); MSSA (0.2); *B. subtilis* (0.3)	KP713630
803B9-1	872	100	*Bacillus sp.*	2	MRSA (0.2)*; B. subtilis* (1.2)	KP713627
803D10-2a	881	*99*	*Lysinibacillus sp.*		MSSA (4.5); MRSA (5.0)	KP713670
803D10-2c	955	99	*Bacillus* sp.	3	MSSA (4.5); MRSA (5.0)	KP713625
803D5	860	99	*Halomonas* sp.		MRSA (10.5)	KP713669
803D9	760	99	*Vibrio* sp.	2	*B. subtilis* (1.0)	KP713585
803E8	913	99	*Pseudoalteromonas* sp.		MRSA (7.5); MSSA (8.5); VRE (4.5); *B. subtilis* (10)	KP713479
803G11	869	99	*Bacillus* sp.		MRSA (6.0)*;* MSSA (4.5); *B. subtilis* (7.5)	KP713626
803G9	811	99	*Pseudoalteromonas* sp.		*B. subtilis* (0.2)	KP713587
803H10	787	99	*Pseudoalteromonas* sp.		MRSA (0.1), *B. subtilis* (0.1)	KP713588
804C6	787	99	*Bacillus cereus*		MRSA (1.25)	KP713589
804D11-1	837	100	*Bacillus* sp.		MRSA (0.1) MSSA (0.1); *B. subtilis* (0.2)	KP713635
804D11-2	897	99	*Lysinibacillus* sp.		MRSA (0.1) MSSA (0.1); *B. subtilis* (0.2)	KP713636
804D11-3	793	99	*Vibrio* sp.		MRSA (0.1) MSSA (0.1); *B. subtilis* (0.2)	KP713637
804D6	802	99	*Bacillus* sp.		MRSA (1.25)	KP713591
804E10	765	99	*Bacillus* sp.		MRSA (0.5)*; B. subtilis* (0.5)	KP713592
804E12	859	99	*Bacillus cereus*		MRSA (2.5)	KP713593
804G3	909	99	*Pseudoalteromonas* sp.		MRSA (0.5)	KP713594
804G9	791	99	*Bacillus megaterium*		*B. subtilis* (8.0)	KP713595
804H10	836	99	*Bacillus* sp.		MRSA (0.1), *B. subtilis* (0.1)	KP713476
805A10	738	99	*Psychrobacter celer*		*B. subtilis* (0.75)	KP713598
805A6	902	99	*Psychrobacter* sp.		*B. subtilis* (0.2)	KP713478
805B1	809	99	*Marinobacter hydrocarbonoclasticus*		*B. subtilis* (0.1)	KP713599
805B12	883	99	*Vibrio* sp.	5	*B. subtilis* (0.5)	KP713600
805C10	737	100	*Alteromonas* sp.		*B. subtilis* (0.1)	KP713603
805C12	771	99	*Vibrio* sp.		*B. subtilis* (0.25)	KP713575
805C7	851	99	*Pseudoalteromonas* sp.		*B. subtilis* (1.5)	KP713601
805E11	570	99	*Shewanella* sp.		*B. subtilis* (0.1)	KP713606
805E12	852	100	*Bacillus* sp.		*B. subtilis* (0.5)	KP713481
805E7	860	99	*Pseudoalteromonas* sp.		*B. subtilis* (4.5)	KP713605
805F10	694	99	*Vibrio* sp.		*B. subtilis* (0.2)	KP713609
805F12	845	99	*Bacillus* sp.		*B. subtilis* (0.1)	KP713576
805F4	817	100	*Marinobacter* sp.		*B. subtilis* (0.1)	KP713607
805H7	1002	99	*Pseudoalteromonas* sp.	11	*B. subtilis* (0.1)	KP713577
806B10	832	99	*Shewanella* sp.		MSSA (>10)	KP713485
806B11	919	99	*Alteromonas* sp.		MSSA (>10)	KP713486
806B12	795	99	*Pseudoalteromonas* sp.		*B. subtilis* (0.2)	KP713487
806C11	816	99	*Alteromonas* sp.		MSSA (>10)	KP713492
806C12	886	99	*Pseudoalteromonas* sp.	2	*B. subtilis* (0.2)	KP713493
806C7	875	99	*Pseudoalteromonas* sp.		*B. subtilis* (0.5)	KP713489
806C9	775	99	*Vibrio* sp.		*B. subtilis* (0.2)	KP713490
806E11	831	99	*Vibrio maritimus*		MSSA (0.1)	KP713618
806F10	624	100	*Vibrio* sp.		MRSA (0.1)*; B. subtilis* (0.2)	KP713631
807A1	965	99	*Vibrio* sp.	13	*B. subtilis* (0.1)	KP713496
807A6	875	99	*Vibrio parahaemolyticus*		*B. subtilis* (0.1)	KP713499
807E3	752	99	*Vibrio* sp.		*B. subtilis* (0.2)	KP713501
807H7	851	99	*Vibrio* sp.		VRE (1.5)	KP713507
807H8	889	99	*Vibrio* sp.		VRE (0.5)	KP713508
808A12	653	99	*Pseudoalteromonas* sp.		MRSA (0.5); *B. subtilis* (0.2)	KP713457
808A7	918	99	*Psychrobacter* sp.		*B. subtilis* (0.5)	KP713456
808B1	918	99	*Pseudoalteromonas* sp.		*B. subtilis* (0.3)	KP713482
808C1	903	99	*Psychrobacter* sp.		*B. subtilis* (0.3)	KP713458
808E1	872	99	*Psychrobacter* sp.		*B. subtilis* (0.1)	KP713459
808F11	624	99	*Pseudoalteromonas* sp.		MRSA (0.2)*; B. subtilis* (0.1)	KP713460
808G11	942	100	*Vibrio* sp.		*B. subtilis* (1.5)	KP713461
808H4	950	99	*Alteromonas* sp.		*B. subtilis* (0.1)	KP713462
814A6	936	99	*Bacillus cereus*		MRSA (1.0); MSSA (2.0)	KP713578
815D3	512	99	*Paracoccus* sp.		*E. coli* (2.0)	KP713579
815G7	834	99	*Pseudoalteromonas* sp.		*B. subtilis* (0.1)	KP713581
816C8	854	99	*Bacillus* sp.		MRSA (2.0) MSSA (1.0); *B. subtilis* (4)	KP713573
823B6-2c	804	99	*Exiguobacterium* sp.		MRSA (0.3) MSSA (1.0); *B. subtilis* (3.5)	KP713622


*Not all isolates initially tested for antibiotic activity are included in this table due to loss of viability during repeated sub-culturing and/or frozen storage or an inability to obtain adequate template or sequence DNA. Test strains include: *Bacillus subtilis* (ATCC 6633 Km resistant), *Enterococcus faecalis* (ATCC 29212), Vancomycin-resistant *Enterococcus* (VRE, ATCC MP-1), Methicillin-sensitive *Staphylococcus aureus* (MSSA, ATCC 29213), Methicillin-resistant *Staphylococcus aureus* (MRSA, ATCC 43300), *Escherichia coli* O157, *Serratia marcescens* PDL100, and *Vibrio shilonii* BAA-91. The closest match and %ID are reported as the most similar bacterial isolates from the NCBI’s GenBank database. Antibacterial spectrum (AB spectrum) was measured as the distance from the edge of the colony (mm) to the end of the zone of inhibition (ZI). Values are reported for inhibition of any tester strain. bp = number of DNA base pairs blast searched. Isolate # illustrates the number of purified isolates with identical sequences and antibiotic production range/spectrum. Accession # = Genbank accession number of the representative bacterial isolate.*

### Antibacterial Screening

**FIGURE 2:**
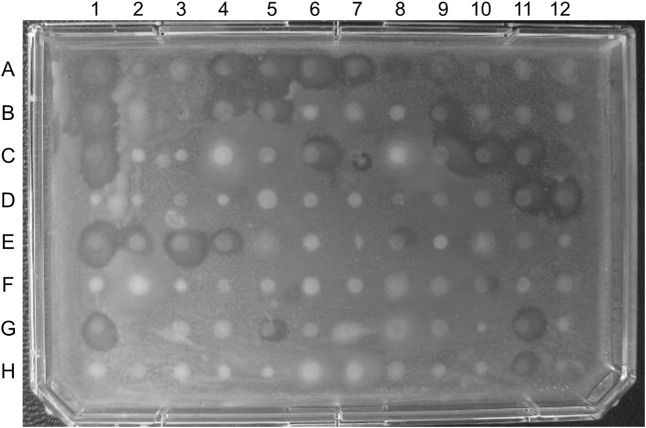
Plate containing 96 of the 323 bacterial isolates cultured from marine Atlantic stingray (*D. sabina)* mucus overlaid with vancomycin-resistant enterococcus (VRE). Zones of inhibition are visible in 30 of the 96 isolates assayed on this plate.

**FIGURE 3:**
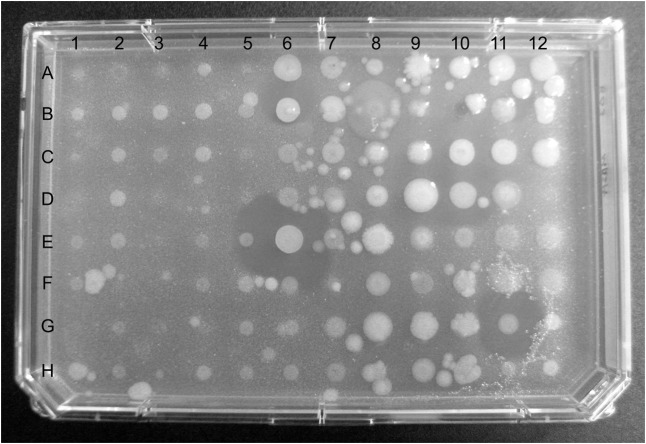
Plate containing 96 of the 960 bacterial strains cultured from cownose ray (*Rhinoptera bonasus*) mucus overlaid with methicillin-resistant *Staphylococcus aureus* (MRSA). Diameters of zones of inhibition in wells E6 and G11 are 7.5 mm and 6.0 mm, respectively.

Analysis of zones of inhibition against pathogenic test strains identified a number of bacterial isolates that displayed antibiotic properties. Examples of pathogen inhibition by mucus-associated isolates are shown in **Figures 2**, **3**. Two hundred out of 960 (21%) *R. bonasus* isolates, 60 of 323 (19%) marine *D. sabina* isolates, 9 of 96 (9%) of freshwater *D. sabina* isolates, 35 of 193 (18%) of *M. hypostoma* isolates and 7 of 288 (2%) of *R. eglanteria* isolates displayed inhibition against at least one test pathogen (**Table 1**).

### Phylogenetic Identification of a Subset of Total Ray Isolates

**Table 2** represents a list of representative antibiotic producing bacterial associates from ray species. Not all isolates initially tested for antibiotic activity were phylogenetically identified due to loss of viability during repeated sub-culturing and/or frozen storage, or an inability to obtain adequate template or sequence DNA. Up to 20% of antibiotic producing isolates did not survive repeated freeze thaw upon cryopreservation and are, therefore, not included in phylogenetic identification attempts. Bacteria associated with the clearnose skate, *R. eglanteria*, were not phylogenetically identified for this study due to loss of strains during long-term storage. Repeated isolates are indicated in **Table 2** and were dereplicated based on identical 16S rDNA sequences and antibiotic spectrum profiles.

#### Freshwater *D. sabina* Bacterial Isolates

Freshwater stingray isolates showed the greatest amount of diversity with 18 genera revealed (data not shown for non-antibiotic producing bacteria) yet had the lowest percentage of antibiotic activities among all the stingray isolates surveyed. Of 96 freshwater *D. sabina* bacterial isolates screened, only 9 (9%) isolates showed antibiotic activities against one or more test strains (**Table 2**). Eight isolates were active against only *E. coli* and consisted mainly of members of the genus *Pseudomonas*, with one *Psychrobacter* and one *Stenotrophomonas* sp. showing similar activities against *E. coli*. One *B. cereus* isolate displayed broad-spectrum activity against *E. coli*, MRSA, MSSA and VRE (**Table 2**). Other non-antibacterial producing isolates making up the culturable flora of freshwater *D. sabina* mucus included *Gordonia*, *Mycobacterium*, *Microbacterium*, *Caulobacter*, *Brevundimonas*, *Chryseobacterium*, *Staphylococcus*, *Psychrobacter*, *Nocardia*, *Bosea*, *Rhizobium*, *Delftia*, *Stenotrophomonas*, *Leucobacter*, *Acinetobacter*, and *Ochrobactrum* spp. (data not included).

#### Marine *D. sabina* Bacterial Isolates

Of 323 marine *D. sabina* bacterial isolates screened, 60 (19%) displayed antibiotic activity (**Table 1**), and only against *B. subtilis* (**Table 2**). Of eight active isolates genetically identified, 16S rDNA sequence analysis revealed 3 bacterial genera including *Vibrio* spp. (*n* = 4), *Photobacterium* spp. (*n* = 3), and a *Staphylococcus* sp. (*n* = 1).

#### *M. hypostoma* Bacterial Isolates

Of 193 *M. hypostoma* bacterial isolates screened, 35 (18%) produced antibiotic activities against one or more test strains (**Table 1**). Of 20 isolates genetically identified, only three bacterial genera were revealed (**Table 2**). Twelve *Vibrio* isolates showed activities against *B. subtilis;* one showed activity against *VRE*; 1 showed activity against both MRSA and *B. subtilis;* and 1 isolate showed no antibacterial activity against any pathogenic strain tested. Ten isolates were identified as *Pseudoalteromonas* species with 4 isolates showing activities against *B. subtilis*, 4 against *V. shilonii*, and 2 showing no activities to any pathogenic strain tested. One *Alteromonas* species showed activity against *B. subtilis* only (**Table 2**).

#### *R. bonasus* Bacterial Isolates

Of 960 bacteria isolated and screened, 200 (21%) were active against one or more test strain (**Table 1**). Eleven different bacterial genera were identified that produce antibacterial compounds, with members of the genera *Bacillus*, *Exiguobacterium*, *Lysinibacillus*, *Vibrio*, and *Pseudoalteromonas* displaying the broadest spectra of activity (**Table 2**). One *Halomonas* sp. (803D5) displayed antibiotic activity against MRSA with a zone of inhibition exceeding 10 mm. *Bacillus* sp. 803E6, displayed large zones of inhibition against MRSA (7.5 mm), MSSA (8.5 mm), VRE (4.5 mm) and *B. subtilis* (10 mm) (**Table 2**). One *Shewanella* sp. (806B10) and one *Alteromonas* sp. (806B11) were active against MSSA with zones of inhibition over 10 mm. Two *Vibrio sp.* demonstrated antibacterial activities against VRE (**Table 2**).

#### Phylogenetic Tree of Antibiotic-Producing Bacteria

**FIGURE 4:**
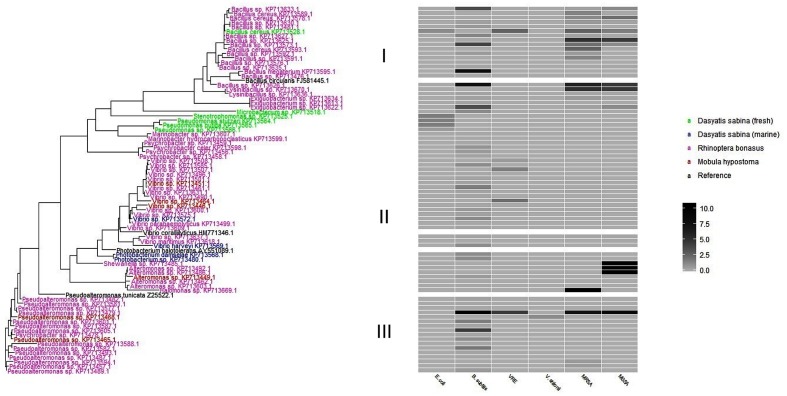
A phylogenetic tree of antibiotic producing bacteria found in this study. Each taxon is colored based on host stingray species. The heatmap to the right represents the zone of inhibition (mm) of candidate strains against test strains. Clusters are designated as I, II, and II to the left of the heat map for discussion purposes.

To better visualize the phylogenetic relatedness of antibiotic producing strains found in this study (**Table 2**) a phylogenetic tree (**Figure 4**) was constructed. In order to address relatedness to other antibiotic-producing bacteria, we included reference sequences from *P. tunicata* strain D2: Z2552, *B. circulans*, FJ581445, *V. coralliilyticus*, HM771346, and *P. halotolerans*, AY551089). *R. bonasus* bacteria were the most abundant isolates genetically identified in this study and formed the basis of the three phylogenetic clusters illustrated in **Figure 4**. *M. hypostoma* isolates clustered within groups II and III and marine *D. sabina* clustered entirely within group II. Isolates from fresh water and marine *D. sabina* did not cluster together. The freshwater *D. sabina* isolates clustered between group I and II with a single isolate positioned within the *R. bonasus* dominated group I *Bacillus* isolates. This fresh water Atlantic stingray isolate, *B. cereus* 845E9, showed inhibition against numerous test strains (*E. coli*, MRSA, MSSA, VRE; **Table 2**). The *R. bonasus* isolates positioned closest to strain 845E9 in **Figure 4** showed similar inhibitory properties against MSSA and MRSA with varying inhibitory properties against other test strains (**Table 2**). *Alteromonas* and *Shewanella* isolates derived from *R. bonasus* produced the strongest zones of inhibition observed against both MSSA and MRSA (heatmap, **Figure 4** and **Table 2**).

### Antibacterial Compound Characterization of a Subset of Broad-Spectrum Antibacterial-Producing Isolates

Eleven strains from *M. hypostoma* and *R. bonasus* were selected for further characterization based on activities against a range of nosocomial pathogens, as well as an ability to survive repeated subculturing and cryopreservation. Compound characterization included hemolytic activity testing and testing for activity in the presence of Proteinase K. Hemolysis tests were performed in order to identify antibiotics with potential toxicity to eukaryotic cells and proteinase *K* tests were used to specifically determine which activities rely on the presence of small peptide antibiotics (**Table 3**). Seven of 9 strains tested from *R. bonasus* were found to be hemolytic. In contrast, both strains isolated from *M. hypostoma* did not display hemolytic activity. The activity of both *M. hypostoma* strains was inhibited by the presence of Proteinase K in antibacterial assays against MRSA and *B. subtilis* suggesting that the active compound is a peptide antibiotic. Antibiotic activities in 3 of 9 *R. bonasus* strains were inhibited by the presence of Proteinase K, suggesting that the active compound(s) in these strains are peptide antibiotics.

**Table 3:** Blood agar and Proteinase K assays performed on a subset of broad-spectrum antibacterial-producing isolates.

Isolate	Zones of inhibition (mm)	Blood Agar (mm)	Proteinase K	Source
803A6	MRSA (7.5); MSSA (8.5); VRE (4.5); *B. subtilis* (10)	Hemolytic (2)	Uninhibited by enzyme	RB
803D102c	MSSA (4.5); MRSA (5.0)	Hemolytic (0.5)	Uninhibited by enzyme	RB
803E6	MRSA (7.5); MSSA (8.5); VRE (4.5); *B. subtilis* (10)	Hemolytic (3)	Uninhibited by enzyme	RB
803G11	MRSA (6.0)*;* MSSA (4.5); *B. subtilis* (7.5)	Not hemolytic	Uninhibited by enzyme	RB
804D4	*B. subtilis* (6); VRE (2.5)	Hemolytic (1)	Inhibited by enzyme	RB
805D11	*B. subtilis* (6.5); MRSA (2); MSSA (1.5);*V. shilonii* (1.0)	Hemolytic (1)	Inhibited by enzyme	RB
806E8	*B. subtilis* (6.5); MRSA (2); MSSA (1.5)	Not hemolytic	Inhibited by enzyme	RB
816C6	*B. subtilis* (5); MRSA (2); MSSA (1)	Hemolytic (0.5)	Uninhibited by enzyme	RB
816C8	MRSA (2.0) MSSA (1.0); *B. subtilis* (4)	Hemolytic (2)	Uninhibited by enzyme	RB
809A9	MRSA (1); MSSA (1); VRE (1)	Not hemolytic	Inhibited by enzyme	MH
809D9	MRSA (1); MSSA (1)	Not hemolytic	Inhibited by enzyme	MH


*Isolates were chosen for further study based on their range of antibiotic activities against human pathogenic test strains and an ability to remain active and viable upon repeated freeze/thaw and subculturing. Zones of inhibition for antibiotic screening are indicated by measuring clearning zones in millimeters (mm). Hemolytic activity was measured on blood agar by measuring zones of clearing in millimeters (mm). Proteinase K assays were utilized to determine if the active compound(s) are peptide antibiotics. The animal source is abbreviated RB for the Cownose Ray *Rhinoptera bonasus* and MH for the Devil Ray *Mobula hypostoma.**

## Discussion

As elasmobranchs display impressive wound healing capabilities, the intent of this study was to survey culturable ray and skate bacterial associates for antibiotic activity with the ultimate goal of identifying a new marine source for novel anti-infective compounds. Results of antibiotic overlays demonstrated that a number of mucus associated bacteria display antibiotic activity against common pathogens. In other studies, protein extracts of the stingray mucus layers have presented similar antibiotic activity (Vennila et al., 2011; Conceição et al., 2012). Collectively over 16% of bacterial isolates of the epidermal mucus from three stingray and one skate species displayed antibiotic activity against one or more pathogenic test strain. Previous studies have illustrated that roughly 3% of bacteria cultured from seawater and up to 13% of bacteria isolated from abiotic or biotic surfaces are inhibitory (Gram et al., 2010). Up to 20% of bacteria isolated from corals produce antibiotic activities (Ritchie, 2006). In the present study, roughly 3% showed either broad-spectrum antibacterial activity or activities against VRE or MRSA, only, suggesting that these isolates, and representative compounds, may be promising candidates for future drug discovery initiatives. Broad-spectrum antibiotic-producing bacterial strains isolated from stingrays, or strains active against important nosocomial pathogens (MRSA and VRE), were identified within 5 different genera including *Bacillus, Vibrio, Exiguobacterium, Lysinibacterium*, and *Pseudoalteromonas.* This information will help in targeting bacterial growth conditions for future antibiotic screens. It is important to note that some small zones of inhibition reported could be caused by factors other than antibiotic production, such as absence of nutrients, changes in pH or other variables that were not measured in this study.

Because of their ability to osmoregulate over a broad range of salinities, both marine and freshwater populations of the Atlantic stingray inhabit Florida waters. This provided a unique opportunity to consider the influence of environment on mucus-associated microbial communities in freshwater and marine *D. sabina*. We phylogenetically identified a large pool of total isolates from both freshwater and marine Atlantic stingrays among both antibiotic-producing and non-antibiotic-producing isolates (**Table 2** and Supplementary Table 1). A predominance of *Vibrio* and *Photobacteria* spp. were associated with marine *D. sabina* (Supplementary Table 1). In comparison we isolated a wide range of bacterial types associated with freshwater *D. sabina*, including members of the genera *Brevundimonas, Psychrobacter, Gordonia, Chryseobacterium, Staphylococcus, Microbacterium, Acinetobacter, Caulobacter, Mycobacterium, Bosea, Nocardia, Rhizobium*, and others (Supplementary Table 1). This is the first research to our knowledge to address a comparison of *D. sabina* bacterial host specificity in fresh vs. marine waters. Other studies have shown host species-specific bacterial associations regardless of differences in environmental locations (Knowlton and Rohwer, 2003; Taylor et al., 2003). Our limited culture-based data suggest a lack of host-driven specificity in Atlantic stingrays that is likely driven by the extreme salinity differences in freshwater vs. marine environments.

Of note is the lack of overlap in antibiotic producing and non-antibiotic producing bacteria associated with freshwater *D. sabina*, where antibiotic producers fall within the genera *Pseudomonas, Stenotrophomonas, Psychrobacter*, and *Bacillus.* Representative active isolates derived from fresh water *D. sabina* were all active against *E. coli* with *Bacillus* strain 845E9 being the only strain with activity against gram-negative *E. coli* as well as gram-positive MRSA, MSSA and VRE (**Table 2**). In contrast, antibiotic-producing bacteria isolated from marine *D. sabina* fall within the genera *Vibrio*, *Photobacterium*, and *Staphylococcus*. These isolates were predominantly active against *B. subtilis* and illustrated a distinct overlap with similar isolates that did not show antibiotic-producing capabilities.

Perhaps as a reflection of their tannic acid river and lake environment, mucus from the freshwater rays is noticeably darker than mucus from marine specimens, with the majority of pigment remaining in the microbe-rich mucus pellet. In addition, in their relatively confined lake environment, freshwater *D. sabina* are exposed to anthropogenic influences of pollution from stormwater runoff and contamination from sewage (Gelsleichter et al., 2006). It is interesting to note that freshwater stingray isolates showed the greatest diversity (with 18 genera revealed among all antibiotic and non-antibiotic producing isolates genetically identified; Supplementary Table 1) and the lowest percentage of antibiotic activities among all the ray isolates surveyed (**Table 1**). That all nine of the isolates from freshwater ray mucus were active against *E. coli* may reflect an adaptive innate immune mechanism driven by beneficial bacterial associates. These data illustrate different bacterial community associations as well as differences in bacterial genera capable of producing antibiotic compounds associated with the epidermal mucus layers of stingrays that have adapted to dramatically different environments. Future efforts using molecular techniques will be necessary to thoroughly address differences in Atlantic stingray bacterial host specificities and potential functional redundancies of freshwater and marine bacterial associates.

The higher total number of bacteria isolated from the cownose ray, *R. bonasus*, is reflected in the higher sample size of 74 individuals from this ray species. In contrast to Atlantic stingray isolates, identification of active isolates from *R. bonasus* revealed that a majority are members of the genera *Bacillus*, *Pseudoalteromonas*, and *Vibrio*, with other active isolates belonging to the genera *Alteromonas*, *Exiguobacterium*, *Psychrobacter*, *Lysinibacillus*, *Halomonas*, *Marinobacter*, *Shewanella*, and *Paracoccus*. With the exception of *Vibrio* spp., different genera are represented among the antibiotic producing isolates from this ray species. This higher apparent diversity is likely due to the higher animal sample size. Overall, although there is apparent overlap in antibiotic producing bacterial species across all ray species analyzed, different isolates were considered unique due to host source and different colony or antibiotic spectra phenotypes and may represent bacteria in normal associations with these animals. Additional biases may include different animal capture methods. Cownose rays and devil rays were collected in natural habitats using a seine net and dip nets to access animals for sampling. While animals were rinsed with sterile seawater prior to mucus sampling, these collection techniques may have removed adhering bacteria while contributing non-associated bacteria to our isolate pools.

The phylogenetic tree shown in **Figure 4** reflects stingray-derived bacterial isolates that display varying degrees of antibiotic activities. As noted above, isolates from fresh water and marine *D. sabina* did not cluster together. Although this study did not address total microbial community differences between hosts, this finding suggests that habitat type can drive culturable host communities even in the same host species. This is likely reflective of the long established inland freshwater versus marine habitats of these Atlantic stingray groups that influences bacterial associates. Marine *Bacillus* species have been shown to produce lipopeptides that are active against a range of gram positive and gram negative pathogenic test strains (Das et al., 2008) which could be a source of activity seen in our active *Bacillus* isolates. *Pseudoalteromonas* species associated with a variety of marine eukaryotes are also known to produce a range of bioactive antagonistic extracellular compounds, including antifouling agents, which may enable them to colonize surfaces (Holmström and Kjelleberg, 1999). Wietz et al. (2010) chemotyped over 300 marine *Vibrionaceae* and *Photobacterium* species revealing the presence of the previously described antibiotics, andrimid (from *Vibrionaceae*) and holomycin (from *Photobacterium*). Andrimid is a broad-spectrum peptide antibiotic originally isolated from a bacterial endosymbiont of a plant-associated arthropod (Fredenhagen et al., 1987). Holomycin also exhibits broad-spectrum activity and belongs to pyrrothine class of antibiotics that acts to interfere with RNA synthesis (Wietz et al., 2010).

Lastly, we have sub-classified active compounds from 11 candidate antibiotic-producing bacterial strains. We selected this subset of active isolates from *R. bonasus* and *M. hypostoma* based on the range of test strains against which they were active, as well as their ability to retain stable activity upon repeated subculture. Proteinase K assays were performed in order to characterize compounds from ray associated bacterial isolates as peptide or non-peptide antibiotics (**Table 3**). Roughly half of the active isolates tested produce peptide antibiotics as the active compound, as assayed based on loss of antibiotic activity in the presence of proteinase K. In addition blood agar assays indicate that compounds produced by the majority of isolates tested, both peptide and non-peptide, are able to lyse blood cells (**Table 3**), indicating a potential toxicity to eukaryotic cells and providing another criteria from which to sub classify and prioritize active strains for future studies.

This study represents an initial search for antibiotic producing bacteria associated with ray and skate species and many isolates will be pursued for drug discovery efforts. Future studies will include molecular determination of a baseline of bacterial associates of elasmobranchs to provide a more comprehensive understanding of the stability of microbial associates across species and habitats. Future work will also contribute to an understanding of the potential role of these associates in the health and wound healing capabilities of elasmobranchs.

## Author Contributions

KR, CL, and CW designed the study. CL and CW collected all epidermal mucus samples. KR, MS, JM, VL, and DM carried out bacterial culturing, antibiotic assays, genetic analysis, and helped prepare tables. CL prepared **Figures 1**–**3**. MS prepared **Figure 4**. KR, JM, MS, and CL drafted the manuscript. All authors read, edited and approved the final manuscript.

## Conflict of Interest Statement

The authors declare that the research was conducted in the absence of any commercial or financial relationships that could be construed as a potential conflict of interest.
