# Supplementary material for: Survey of Antibiotic-producing Bacteria Associated with the Epidermal Mucus Layers of Rays and Skates
Source: Front Microbiol. 2017 Jul 5;8:1050. doi: 10.3389/fmicb.2017.01050 (PMC5496964; doi:10.3389/fmicb.2017.01050)
Supplement: Supplementary file 1 [file Data_Sheet_1.docx]

## Supplementary Table 1. Partial list bacteria isolated from the mucus of rays that do not exhibit antibiotic activities. Not all isolates were chosen for genetic ID. The closest match and % ID are reported as the most similar bacterial isolates from the NCBI’s GenBank database. bp = number of DNA base pairs blasted. Accession # = Genbank accession number of bacterial isolate.

| **Strain** | **bp** | **% ID** | **Strain Identification** | | **Accession #** |
| --- | --- | --- | --- | --- | --- |
| **Freshwater Atlantic Stingray, *Dasyatis sabina*** | | | | | |
| 845 A2A | 770 | 100 | *Gordonia terrae* | | KP713641 |
| 845 A2B | 820 | 99 | *Mycobacterium* sp. | | KP713642 |
| 845 A3 | 780 | 99 | *Microbacterium* sp. | | KP713668 |
| 845 A4 | 832 | 99 | *Caulobacter* sp. | | KP713643 |
| 845 A5 | 623 | 100 | *Caulobacter* sp. | | KP713644 |
| 845 B11 | 804 | 99 | *Brevundimonas vesicularis* | | KP713522 |
| 845 B12 | 860 | 99 | *Chryseobacterium* sp*.* | | KP713523 |
| 845 B5A | 858 | 99 | *Staphylococcus pasteuri* | | KP713519 |
| 845 B5B | 815 | 99 | *Staphylococcus pasteuri* | | KP713520 |
| 845 B6 | 654 | 99 | *Psychrobacter alimentarius* | | KP713534 |
| 845 B7 | 662 | 98 | *Psychrobacter* sp. | | KP713535 |
| 845 B8 | 902 | 99 | *Brevundimonas vesicularis* | | KP713521 |
| 845 C12 | 790 | 99 | *Psychrobacter* sp. | | KP713524 |
| 845 C1A | 847 | 99 | *Brevundimonas* sp. | | KP713645 |
| 845 C1B | 772 | 99 | *Microbacterium* sp. | | KP713646 |
| 845 C3 | 691 | 100 | *Nocardia* sp. | | KP713638 |
| 845 C4 | 580 | 99 | *Gordonia bronchialis* | | KP713639 |
| 845 C6A | 588 | 100 | *Bosea lupini* | | KP713647 |
| 845 C6B | 789 | 97 | *Bosea* sp. | | KP713648 |
| 845 C7 | 644 | 99 | *Mycobacterium fortuitum* | | KP713649 |
| 845 C9A | 604 | 99 | *Gordonia terrae* | | KP713650 |
| 845 D1 | 679 | 99 | *Acinetobacter* sp. | | KP713536 |
| 845 D10 | 900 | 99 | *Rhizobium* sp. | | KP713666 |
| 845 D11 | 600 | 99 | *Brevundimonas* sp. | | KP713539 |
| 845 D2 | 899 | 99 | *Delftia tsuruhatensis* | | KP713537 |
| 845 D8 | 811 | 100 | *Bosea* sp. | | KP713640 |
| 845 D9 | 752 | 99 | *Pseudomonas xanthomarina* | | KP713538 |
| 845 E12 | 896 | 99 | *Chryseobacterium hominis* | | KP713529 |
| 845 E2 | 621 | 99 | *Gordonia polyisoprenivorans* | | KP713526 |
| 845 E3 | 721 | 100 | *Brevundimonas* sp. | | KP713527 |
| 845 F1 | 544 | 100 | *Gordonia* sp. | | KP713530 |
| 845 F3 | 824 | 99 | *Leucobacter alluvii* | | KP713531 |
| 845 F7 | 612 | 99 | *Acinetobacter* sp. | | KP713541 |
| 845 F8 | 548 | 97 | *Acinetobacter* sp. | | KP713667 |
| 845 G1 | 653 | 98 | *Acinetobacter* sp. | | KP713542 |
| 845 G11 | 620 | 99 | *Chryseobacterium* sp. | | KP713545 |
| 845 G12 | 822 | 99 | *Microbacterium oxydans* | | KP713532 |
| 845 G4 | 688 | 98 | *Psychrobacter* sp. | | KP713543 |
| 845 G9 | 824 | 99 | *Chryseobacterium* sp. | | KP713544 |
| 845 H2 | 862 | 98 | *Ochrobactrum anthropi* | | KP713546 |
| 845 H3 | 869 | 98 | *Chryseobacterium* sp. | | KP713547 |
| 845 H5 | 870 | 98 | *Psychrobacter piscatorii* | | KP713548 |
| **Marine Atlantic Stingray, *Dasyatis sabina*** | | | | | |
| 846 A1 | 863 | 99 | | *Psychrobacter celer* | KP713549 |
| 846 A7 | 1035 | 99 | | *Vibrio proteolyticus* | KP713533 |
| 846 A12 | 772 | 100 | | *Vibrio proteolyticus* | KP713550 |
| 846 B9 | 865 | 99 | | *Vibrio sp.* | KP713551 |
| 846 B10 | 929 | 99 | | *Vibrio sp.* | KP713552 |
| 846 E10 | 806 | 100 | | *Photobacterium damselae* | KP713553 |
| 846 E11 | 827 | 98 | | *Vibrio harveyi* | KP713554 |
| 846 E12 | 739 | 99 | | *Photobacterium damselae* | KP713555 |
| 846 G1 | 841 | 100 | | *Vibrio proteolyticus* | KP713556 |
| 846 G2 | 849 | 99 | | *Exiguobacterium sp.* | KP713557 |
| 846 H1 | 734 | 100 | | *Vibrio alginolyticus* | KP713558 |
| 846 H10 | 895 | 99 | | *Photobacterium damselae* | KP713559 |
| 846 H11 | 873 | 99 | | *Photobacterium damselae* | KP713560 |
| **Atlantic Devil Ray, *Mobula hypostoma*** | | | | | |
| 809 C9 | 889 | 99 | | *Pseudoalteromonas* sp. | KP713470 |
| 809 D5 | 906 | 99 | | *Pseudoalteromonas* sp. | KP713483 |
| 810 E1 | 937 | 99 | | *Vibrio* sp. | KP713445 |
| **Cownose Ray, *Rhinoptera bonasus*** | | | | | |
| 803B10-1 | 844 | 99 | | *Psychrobacter* sp. | KP713628 |
| 803B10-2 | 871 | 99 | | *Bacillus* sp. | KP713617 |
